# Aroma metabolism during the low-temperature storage of Hami melons: volatile odor-active compounds, precursors, and enzyme activity

**DOI:** 10.3389/fnut.2026.1775610

**Published:** 2026-03-30

**Authors:** Xiangshuai Hao, Bo Peng, Chunhui Shan, Wanqin Liao, Mengyao Yang, Ming Ning, Qin Zhang, Fengxian Tang

**Affiliations:** 1 Engineering Research Center of Storage and Processing of Xinjiang Characteristic Fruits and Vegetables, Ministry of Education, School of Food Science, Shihezi University, Shihezi, Xinjiang, China; 2 Key Laboratory of Processing and Quality and Safety Control of Specialty Agricultural Products (Co-construction by Ministry and Province), Ministry of Agriculture and Rural Affairs, School of Food Science, Shihezi University, Shihezi, Xinjiang, China; 3 Key Laboratory for Food Nutrition and Safety Control of Xinjiang Production and Construction Corps, School of Food Science, Shihezi University, Shihezi, Xinjiang, China

**Keywords:** amino acids, aroma, carotenoids, fatty acids, Hami melon, low-temperature storage

## Abstract

To explore the regulatory effect of low-temperature storage on the aroma quality of Hami melons, this study examined the dynamic changes in their aroma precursors, enzyme activities, gene expression profiles, and odor-active compounds content following storage at 3 °C. The low-temperature group exhibited significantly lower levels of primary odor-active compounds—including esters, alcohols, and aldehydes—than the ambient-temperature group, with the greatest disparity observed on day 6 (1211.33 μg/kg). Odour activity value analysis revealed that (E)-2-nonene (609.49), *β*-ionone (434.00), and ethyl 3-methylbutyrate (184.82) contributed most significantly to the aroma of Hami melons. Notably, low-temperature storage markedly reduced the activity of ACSL, BCAT, ADH, AAT, and CCD7 while also downregulating the genes encoding these enzymes, including *CmADH*, *CmBCAT*, and *CmACSL*. Among these enzymes, CCD7 exhibited the greatest difference in activity between the low- and ambient-temperature groups at 18 d of storage (81.04 U/L). Concurrently, low temperature was found to markedly inhibit the degradation of fatty acids, amino acids, and *β*-carotene in Hami melons. Linoleic acid and palmitic acid reached peak concentrations at 18 and 12 d of storage (519.16 mg/kg and 537.70 mg/kg, respectively), while the levels of amino acids and carotenoids remained generally lower than those of fatty acids (0–20 mmol/kg). Correlation analysis demonstrated significant associations between the activities of enzymes such as HPL, ADH, and BCAT and the levels of both aroma precursors and final odor-active compounds. This study provides a theoretical basis for optimising post-harvest storage methods and maintaining flavour quality in Hami melons.

## 1 Introduction

Hami melon (*Cucumis melo* L.) belongs to the *Cucurbitaceae* family and is a renowned traditional product of Xinjiang. Known for its crunchy flesh, sweet taste, unique flavour, and rich nutrient content, Hami melon is highly popular among both domestic and international consumers. Hence, it has become one of the world’s most important and widely cultivated crops (1). Hami melon is a typical climacteric fruit, and its harvest period is centralised to periods of high temperature. Moreover, this fruit shows vigorous post-harvest metabolism and poor storage resistance. Hence, low-temperature storage has emerged as a common practice to extend the shelf life of Hami melon.

Low-temperature storage is commonly used to delay fruit ripening and reduce post-harvest decay, but prolonged storage at low temperatures can impact the aroma quality of fruit. In oranges, lemons, strawberries, peaches, and other fruit, the synthesis of volatile compounds is inhibited during low-temperature storage, and the content of volatile compounds varies based on the range of low temperatures and ripening stages (2–6). The final aroma of fruit arises from the interactions among multiple volatile compounds (7) and is an important indicator of flavour and quality (8–13). Fruit aroma depends on the low-molecular-weight volatile compounds produced during ripening (14). The primary volatile compounds in fruit are esters, alcohols, aldehydes, and ketones (9, 11, 15), which are generated through fatty acid metabolism, amino acid metabolism, terpene synthesis, and other metabolic pathways. Notably, a large variety of aroma precursors and functional enzymes are involved in these metabolic reactions.

To date, approximately 300 volatile compounds—including esters, alcohols, aldehydes, and ketones—have been identified in various melon varieties (1, 16, 17). Among them, esters have emerged as the predominant components contributing to the characteristic aroma of melon fruit (18). Compounds such as esters, alcohols, and aldehydes are primarily derived from the cleavage of secondary metabolites such as fatty acids, amino acid derivatives, carotenoids, and sesquiterpenes (19), In a previous study, 102 volatile compounds were identified in melon fruit, including 40 esters, 25 alcohols, 23 aldehydes, nine ketones, and five other compounds (20). However, the specific mechanisms underlying the generation of these volatile compounds in Hami melon, as well as the changes they undergo during storage, remain unclear and warrant further in-depth exploration.

Building upon investigations into the types and concentrations of odor-active compounds in Hami melons under low-temperature storage conditions, this study explored the dynamics of their aroma precursors, key enzymes, and gene expression profiles, with a special focus on relevant metabolic pathways. Through correlation analysis, the study further examined the potential relationships among aroma precursors, key enzymes, and odor-active compounds in Golden Queen Hami melons. Overall, the study aimed to provide a theoretical basis for enhancing the aroma quality of melons by elucidating the impact of these changes on their aroma characteristics.

## 2 Materials and methods

### 2.1 Experimental materials and processing conditions

Golden Queen Hami melon (*Cucumis melo* L. var. inodorus Jacq) was harvested from a farm in 121 Corps, Shihezi City, Xinjiang at commercial maturity (about 13% SSC). Fruit of uniform shape, size, and colour that were free from physical damage and pests were picked and stored at 3 °C ± 0.5 °C (low temperature, LT) or 21 °C ± 0.5 °C (room temperature, RT). Sampling was performed after 0, 6, 12, 18, and 24 d, with three replicates examined per time point. The collected fruit samples were cut into small pieces, frozen with liquid nitrogen, and stored at −80 °C for all subsequent experiments.

### 2.2 HS-SPME-GC–MS and odour activity value (OAV) analysis

The odor-active compounds in Hami melons were identified based on the method reported by Peng et al. (21), with appropriate modifications. First, 5 g of fruit was weighed and added to a 20 mL headspace vial along with 1.5 g NaCl and 20 μL 40.95 μg/mL 2-octanol (internal standard). After mixing and 30 min of equilibration, sample extraction was performed for another 30 min. The sampler was inserted into the gas chromatography (GC) injector and desorbed at 250 °C for 5 min before analysis using GC coupled with mass spectrometry (GC–MS) (7890-5977A, Agilent Technologies, USA). An HP-INNOWAX (30 m × 0.25 mm × 0.25 μm) column was used, and He was selected as the carrier gas at a flow rate of 1 mL/min. The temperature programme was as follows: 40 °C (held for 3 min), ramped at 4 °C/min to 88 °C (held for 1.5 min), ramped at 2 °C/min to 110 °C (held for 1.5 min), ramped at 4 °C/min to 200 °C (held for 2 min), and finally ramped at 8 °C/min to 240 °C (held for 3 min). The ion source temperature was set at 230 °C, with an electron energy of 70 eV and a mass scan range of 35–400 amu. Odor-active compounds were identified by comparing their retention indices and mass spectra against the NIST 2017 database. According to the Metabolomics Standards Initiative (MSI), the level of compound identification is clearly defined as Level 2.

The content of each compound was determined using 2-octanol as the internal standard. The volatile compounds were quantified without considering response factors and recovery rates, as shown below:


$$C_{X}=\frac{A_{x}}{A_{s}*m_{x}}*m_{s}$$

Among them, C_x_ represents the concentration of the target compound *x*, *A_x_* represents the peak area of compound *x*, A_s_ represents the peak area of the internal standard s, m_x_ represents the sample mass (Kg), and m_s_ represents the mass of the internal standard *s* (*μ*g).

The OAV was calculated as the ratio between the odor-active compounds concentration and the threshold concentration.

### 2.3 Analysis of fruit aroma metabolism-related enzyme activities

First, 0.5 g of fruit pulp was mixed with 4.5 mL of extraction medium (0.05 mol/L Tris–HCl) and homogenised on ice. The homogenate was centrifuged at 4 °C and 5,000 × *g* for 20 min, and the supernatant was used to measure enzyme activity. The activities of long-chain acyl-CoA synthetase (ACSL), lipoxygenase (LOX), hydroperoxide lyase (HPL), alcohol dehydrogenase (ADH), alcohol acyltransferase (AAT), branched-chain amino acid transferase (BCAT), and carotenoid-cleaving dioxygenase7 (CCD7) were determined using commercial plant ELISA kits (Shanghai Youxuan Biotechnology Co., Ltd).

### 2.4 Real-time quantitative PCR (qRT-PCR)

Total RNA was extracted using the MolPure® Plant RNA Kit [Yeasen Biotechnology (Shanghai) Co., Ltd.] based on the manufacturer’s instructions. Primers targeting selected genes were designed using Primer Premier 5.0 software, as detailed in Supplementary Table S1. The qRT-PCR was performed using the SYBR Green Pro Taq HS Pre-mixed qPCR Kit (Hunan Accurate Bioengineering Co., Ltd.) on a QuantStudio™ 6 Flex System (ABI, USA). The temperature programme was as follows: 30 s of pre-denaturation at 95 °C, followed by 40 cycles of 5 s of denaturation at 95 °C and 30 s of extension at 60 °C. The relative expression levels of each target gene were calculated using the 2^−ΔΔCt^ method (22). *CmUBIep* served as the internal reference gene.

### 2.5 Fatty acid analysis

Fatty acid analysis was performed as described previously (8), with slight modifications. First, 6 g of each Hami melon sample was pulverised into a powder and mixed with 7.5 mL of a 6.7% (m/m) sodium sulfate solution and 15 mL of an isopropanol and hexane mixture (2:3 v/v). After vortexing for 2 min, the mixture was centrifuged at 5000 × *g* for 10 min and the supernatant was collected. The residue remaining after centrifugation was extracted for the second time, and the supernatant was once again collected. The two supernatants were combined, and the fatty acid extract was obtained by blowing off the solvent with nitrogen. Then, 3 mL of a sulfuric acid, toluene, and methanol mixture (2:10:88, v/v) was added to the fatty acid extract. The mixture was placed in an 80 °C water bath for 1 h, and subsequently, 1 mL of heptane was added after cooling. After mixing, the upper layer of the liquid was transferred to a centrifuge tube, and 0.2 *g* of anhydrous sodium sulfate was added to absorb the residual water. The extract was filtered using a 0.22 μm organic membrane and added to GC vials to examine the fatty acid content. A DM-Wax column (30 m × 0.25 mm × 0.25 μm) was selected, with the carrier gas being nitrogen (1 mL/min) at an injection volume of 1 μL (without shunt). The temperature of the injection port was set to 220 °C, and the temperature of the column box was kept at 140 °C for 2 min, increased to 220 °C at a rate of 4 °C/min, maintained at 220 °C for 10 min, and then increased to 250 °C for 3 min.

### 2.6 Amino acid analysis

Amino acid analysis was performed as described by Majidano and team, with minor modifications (23). First, 5 g of each Hami melon sample was added to 5 mL of 6 M HCl and heated in a screw-cap vial at 110 °C for 24 h. The mixture was cooled and centrifuged at 3000 × *g* for 20 min. Then, the clarified supernatant was separated, and the residue was washed with 1 mL of deionised water. The solvent was blown off with nitrogen, and the residue was dissolved in water and diluted to a volume of 10 mL. Subsequently, 3 mL of the solution was used for amino acid analysis. The GC analysis procedure was as follows: a DM-Wax column (30 m × 0.25 mm × 0.25 μm) was used; the split ratio was 10:1 v/v; the injector and detector temperatures were 270 °C and 280 °C, respectively; and the column temperature was first maintained at 100 °C for 2 min and then increased to 250 °C at a rate of 20 °C/min. The nitrogen flow rate was 3 mL/min, and the flame ionisation detection (FID) flow rate was fixed as 45 mL/min for nitrogen, 40 mL/min for hydrogen, and 250 mL/min for air.

### 2.7 *Β*-Carotene analysis

β-carotene analysis was performed as described previously (24), with minor modifications. First, 5 g of each frozen Hami melon sample was ground into a powder and mixed with 20 mL of a hexane–ethanol mixture containing 0.01% 2,6-di-tert-butyl-p-cresol (3:4, v/v), sealed, and incubated at 4 °C in the dark with shaking for 20 h. After centrifugation at 4 °C and 5,000 × *g* for 20 min, the supernatant was collected, and the solvent was blown off using nitrogen. The sample was solubilised in 5 mL of n-hexane. Hexane containing 0.01% 2,6-di-tert-butyl-p-cresol was used as the reference solution, and the absorbance was measured at 446 nm using a UV–visible spectrophotometer. The content of β-carotene was calculated using a standard curve and expressed as μg/g.

### 2.8 Statistical analyses

Analysis of variance (ANOVA) and Correlation analyses were performed using IBM SPSS Statistics 26 software. Plots were generated using Origin 2024 software. Differences with *p*-values <0.05 or <0.01 were considered statistically significant.

## 3 Results

### 3.1 Odor-active compounds detected during low-temperature storage

**Figure 1:**
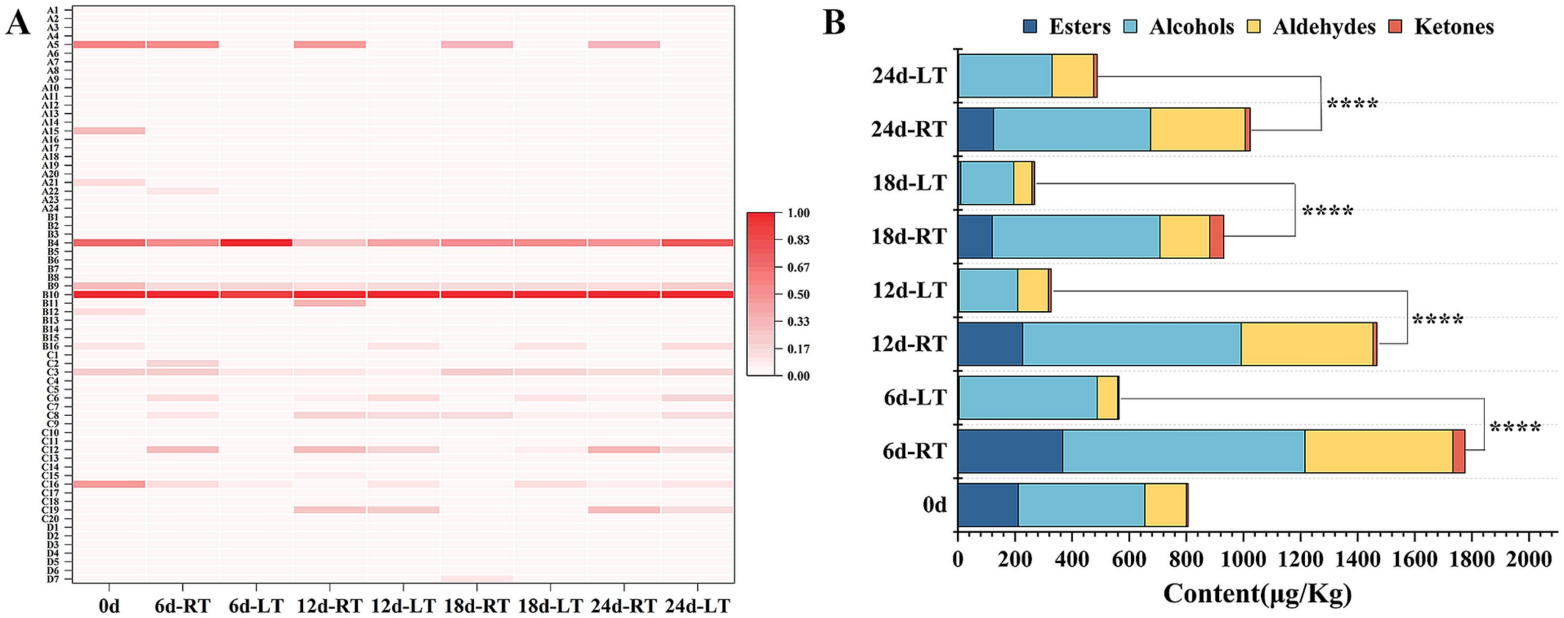
Changes in the content of different odor-active compounds **(A)** and total content **(B)** during storage. *, **, ***, and **** indicate significant difference at *p* < 0.05, *p* < 0.01, *p* < 0.001 and *p* < 0.0001.

As shown in Figure 1A, 67 odor-active compounds in total were identified under both ambient- and low-temperature storage conditions. These included 24 esters (coded A1 to A24), 16 alcohols (coded B1 to B16), 20 aldehydes (coded C1 to C20), and 7 ketones (coded D1 to D7) (Supplementary Table S2). Under ambient temperature conditions, the total odor-active compounds content exhibited an “increase–decrease–increase” trend (Figure 1B), peaking at 1775.56 μg/kg on day 6 before gradually declining to 930.98 μg/kg by day 18. In contrast, under low-temperature conditions, the total odor-active compounds content decreased continuously between days 0 and 18 before rising again on day 24. Furthermore, the total content of odor-active compounds consistently remained lower during low-temperature storage than during ambient storage.

To identify characteristic odor-active compounds throughout the storage period, 20 compounds with an OAV greater than 1 were screened based on GC–MS analysis (Table 1). Among these, ethyl acetate, (E,Z)-3,6-nonadien-1-ol, (6Z)-nonen-1-ol, (Z)-6-nonenal, nonanal, (E,Z)-2,6-nonadienal, (E)-2-nonene, and *β*-ionone were found to contribute significantly to the overall aroma of Hami melons during storage. Meanwhile, ethyl 3-methylbutanoate (OAV 184.82) and ethyl 2-methylpropanoate (OAV 110.04) imparted pronounced fruity notes to Hami melons stored at ambient and low temperatures for 18 d, respectively. Additionally, (E,Z)-3,6-Nonadien-1-ol (OAV 89.48) and (E,Z)-2,6-Nonadienal (OAV 176.97) emerged as the key compounds driving the green aroma of Hami melons stored at ambient temperature for 6 d; β-Pyrone, imparting floral notes, persisted throughout storage and reached peak levels (OAV 434.00) after 18 d at ambient temperature. Overall, low temperature was found to suppress the OAV of melon odor-active compounds, especially esters.

**Table 1:** Tentative identification of principal odor-active compounds during storage (OAV > 1).

Compounds	Thresholds^a^ (μg/Kg)	RT-OAV					LT-OAV					Fragrance properties^b^
		0 d	6 d	12 d	18 d	24 d	0 d	6 d	12 d	18 d	24 d	
Esters												
Ethyl acetate	5.000	23.24	51.65	39.58	22.23	22.06	23.24	<1	<1	<1	<1	Ethereal
Ethyl 3-methylbutanoate	0.010	<1	20.58	<1	184.82	<1	<1	<1	<1	<1	<1	Fruity
Ethyl 2-methylpropanoate	0.020	<1	<1	<1	<1	<1	<1	<1	<1	110.04	<1	Fruity
Ethyl hexanoate	5.000	<1	1.92	1.41	<1	<1	<1	<1	<1	<1	<1	Fruity
Methyl 2-methylbutanoate	0.250	<1	8.71	<1	<1	<1	<1	<1	2.90	<1	<1	Fruity
Alcohols												
(E,Z)-3,6-Nonadien-1-ol	3.000	45.12	89.48	38.70	58.73	55.02	45.12	77.22	17.35	18.00	39.21	Green
1-Nonanol	45.500	1.31	1.74	1.26	1.19	<1	1.31	<1	<1	<1	<1	Floral
(6Z)-Nonen-1-ol	1.000	21.78	11.43	12.37	<1	<1	21.78	<1	12.56	9.64	20.69	Melon
Aldehydes												
Hexanal	5.000	<1	17.06	<1	2.91	<1	<1	<1	1.06	<1	<1	Green
Acetaldehyde	25.100	1.61	4.43	1.57	2.80	2.02	1.61	1.01	<1	<1	1.04	Ethereal
(Z)-6-Nonenal	1.000	3.00	57.09	31.99	8.36	21.50	3.00	<1	15.37	9.31	26.25	Melon
Nonanal	1.100	<1	45.83	74.62	44.44	18.90	<1	6.12	15.12	5.47	21.80	Aldehydic
(E,Z)-2,6-Nonadienal	0.800	6.83	176.97	159.69	22.01	143.62	6.83	15.06	25.05	7.66	28.68	Green
Heptanal	2.800	1.28	2.26	1.90	<1	<1	1.28	<1	<1	<1	<1	Green
(Z)-2-Nonenal	0.020	<1	<1	42.97	<1	<1	<1	<1	65.02	120.06	<1	Fatty
(E)-2-Nonenal	0.190	<1	<1	609.49	<1	505.01	<1	35.23	132.83	25.84	116.96	Fatty
Octanal	0.587	<1	7.90	<1	<1	2.21	<1	<1	<1	<1	<1	Aldehydic
Ketones												
(E)-β-Ionone	0.007	21.73	200.21	140.84	434.00	208.51	21.73	46.05	74.81	57.06	77.36	Floral
Acetoin	14.000	<1	1.39	<1	<1	<1	<1	<1	<1	<1	<1	Buttery
Furaneol	22.300	<1	<1	<1	1.65	<1	<1	<1	<1	<1	<1	Caramellic

a The odor threshold was determined based on the research of L. J. van Gemert (2011). The study compiled data on odor thresholds in air, water and other media.

b The aromatic characteristics were determined by reference to chemical book (https://www.chemicalbook.com).

### 3.2 Effect of low-temperature storage on the activity of key aroma-producing enzymes

**Figure 2:**
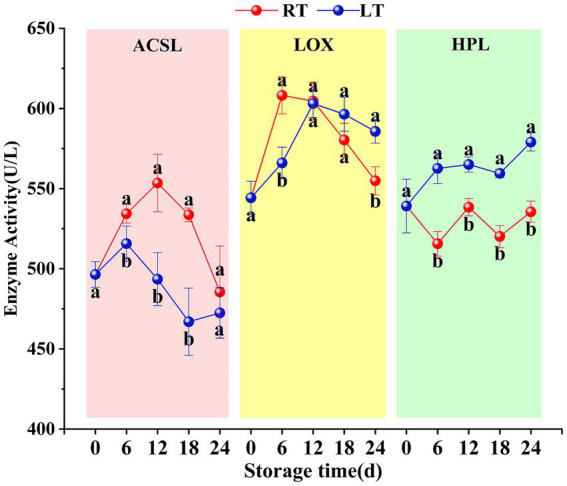
Changes in ACSL, LOX, and HPL activities during storage. Vertical bars represent the standard deviation (*n* = 3). Different letters on the same-day data point represent the significant differences between data (*p* < 0.05).

At ambient temperature, the enzyme activities of both ACSL and LOX in Hami melons exhibited an initial increase, followed by a decline. ACSL activity peaked at 553.52 ± 17.88 U/L on the 12th day of storage, while LOX activity reached its maximum of 608.23 ± 11.47 U/L on the 6th day. HPL enzyme activity, however, fluctuated throughout the storage period. At low temperature, ACSL activity was significantly lower than that at ambient temperature, with a peak activity of 515.71 ± 10.94 U/L observed on day 6. LOX enzyme activity followed similar overall trends at low and ambient temperatures, but it was lower at low temperatures prior to day 12 and higher at ambient temperatures thereafter (Figure 2). Meanwhile, HPL enzyme activity was significantly higher at low temperature than at ambient temperature (Figure 2). Overall, ACSL enzyme activity exhibited a downward trend at low temperatures, whereas LOX and HPL enzyme activities showed an upward trend.

**Figure 3:**
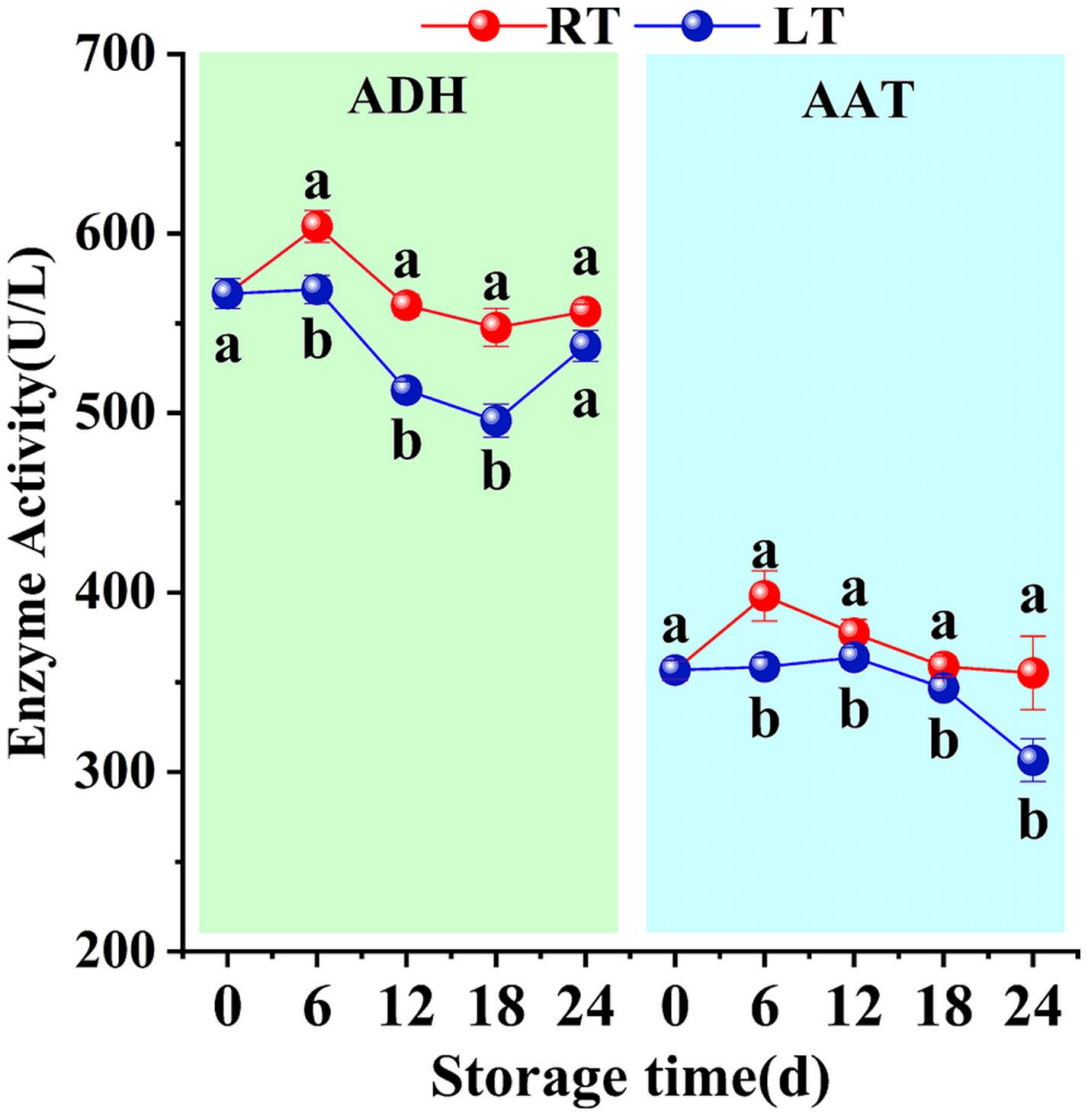
Changes in ADH and AAT activities during storage. Vertical bars represent the standard deviation (*n* = 3). Different letters on the same-day data point represent the significant differences between data (*p* < 0.05).

At ambient temperature, the activity of AAT in Hami melon fruit exhibited an initial increase followed by a decline, whilst the activity of ADH fluctuated. Both ADH and AAT enzyme activities reached peak levels on day 6 (603.97 ± 8.81 U/L and 398.15 ± 14.07 U/L, respectively). Moreover, although ADH and AAT enzyme activities showed consistent overall trends at low and ambient temperatures, they remained significantly lower at low temperature than at ambient temperature (Figure 3).

**Figure 4:**
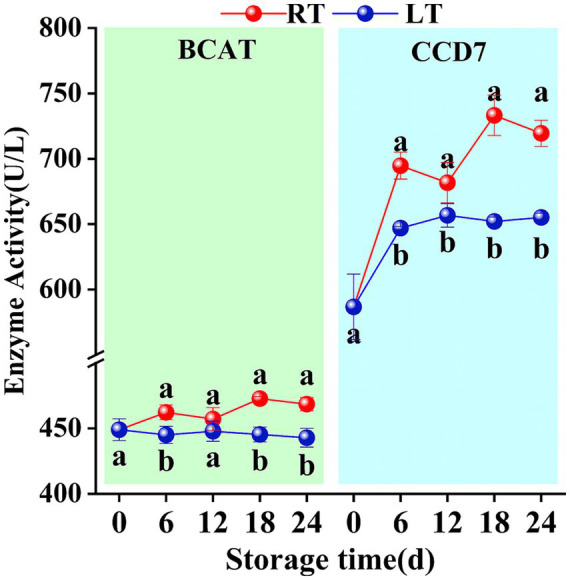
Changes in BCAT and CCD7 activities during storage. Vertical bars represent the standard deviation (*n* = 3). Different letters on the same-day data point represent the significant differences between data (*p* < 0.05).

At ambient temperature, BCAT activity in Hami melon fruit exhibited an overall upward trend, increasing from 449.03 U/L on day 0 to 468.58 U/L on day 24 (Figure 4). Meanwhile, CCD7 enzyme activity showed the most pronounced increase (an overall rise of 23%) (Figure 4). Under low-temperature storage conditions, both BCAT and CCD7 enzyme activities remained lower than those in the ambient temperature group, and changes in activity were also more gradual in this group.

### 3.3 Effect of low-temperature storage on the expression of genes encoding key enzymes

**Figure 5:**
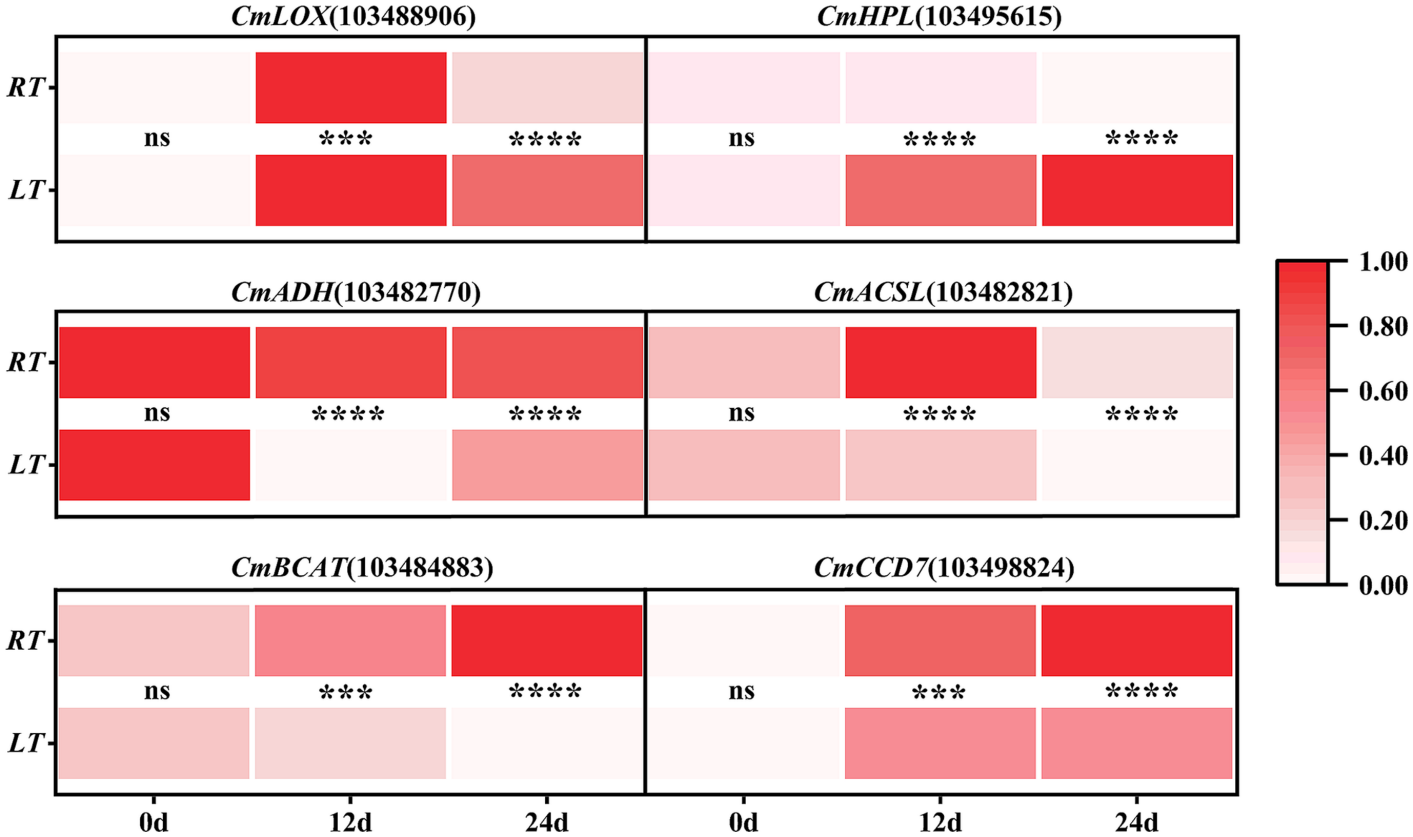
Changes in the expression of key enzyme genes for cantaloupe aroma during storage. *, **, ***, and **** indicate significant difference at *p* < 0.05, *p* < 0.01, *p* < 0.001, and *p* < 0.0001.

The expression trends of *CmLOX* in the ambient temperature group mirrored those of *CmACSL*, exhibiting a dynamic pattern involving an initial increase followed by a decline. In contrast, *CmHPL* expression exhibited a sustained decrease, whereas *CmADH* levels remained consistently high. Meanwhile, *CmBCAT* and *CmCCD7* expression increased as storage was prolonged. Low-temperature treatment inhibited the expression of several genes encoding key aroma-related enzymes. The trends of *CmLOX* and *CmCCD7* expression were consistent between the low- and ambient-temperature groups, though actual *CmCCD7* expression levels remained lower in the former. Meanwhile, *CmHPL* expression levels increased continuously and remained higher in the low-temperature group than in the ambient-temperature group. *CmADH* expression fluctuated, decreasing initially before increasing thereafter. Additionally, *CmACSL* and *CmBCAT* expression levels decreased continuously throughout the storage period. Interestingly, the expression levels of all genes were generally lower in the low-temperature group than in the ambient-temperature group at all time points throughout the storage period. However, consistent with the trends in their enzyme activities, two notable exceptions were *CmLOX* and *CmHPL* (Figure 5).

### 3.4 Effect of low-temperature storage on aroma precursor content

#### 3.4.1 Fatty acid content

During the storage of Hami melons, two saturated fatty acids—palmitic acid and stearic acid—were detected, along with four unsaturated fatty acids (i.e., linoleic acid, linolenic acid, oleic acid, and palmitoleic acid).

**Figure 6:**
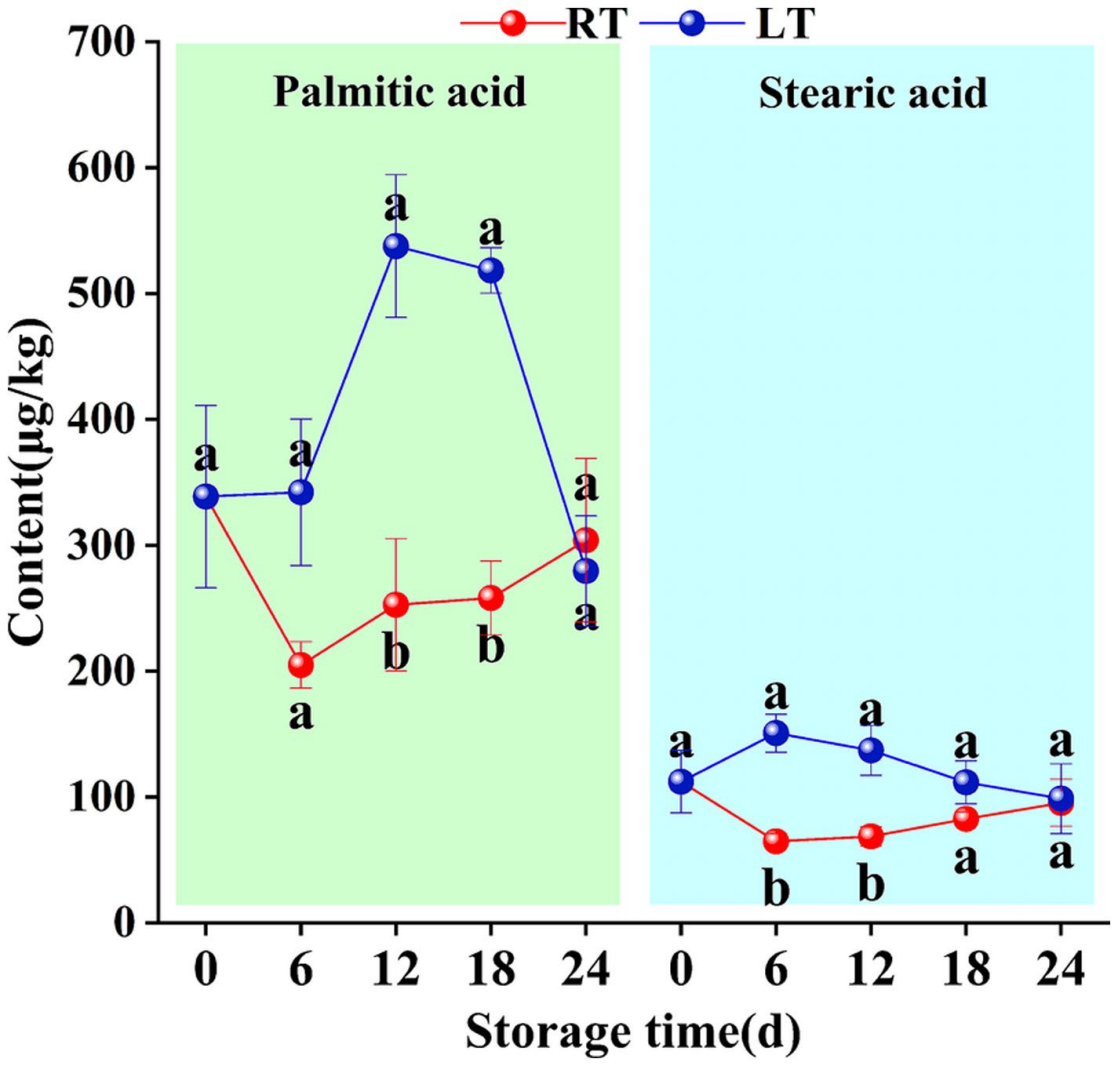
Changes in content of palmitic acid and stearic acid during storage. Vertical bars represent the standard deviation (*n* = 3). Different letters on the same-day data point represent the significant differences between data (*p* < 0.05).

Under ambient conditions, palmitic acid and stearic acid exhibited an initial decline followed by an increase, with both reaching their lowest concentrations on day 6 (204.91 mg/kg and 64.88 mg/kg, respectively). Under low-temperature conditions, the levels of these compounds showed an initial increase, followed by a decline, with peak values detected on day 12 (537.70 mg/kg) and day 6 (150.77 mg/kg), respectively. During low-temperature storage, the content of all saturated fatty acids in Hami melons (except palmitic acid on day 24) was higher than that during ambient-temperature storage. On day 24, the palmitic acid content was 24.38 mg/kg lower under low-temperature conditions than at ambient temperature, with the rate of decline reaching 46.07% during days 18–24 (Figure 6).

As shown in Figure 6, under low-temperature conditions, the content of linoleic acid, linolenic acid, and palmitic acid initially increased in Hami melons before decreasing thereafter. However, the content of linoleic acid and palmitic acid was the highest on day 18 (519.16 mg/kg and 153.05 mg/kg, respectively), while that of linolenic acid peaked at 346.93 mg/kg on day 12. In addition, oleic acid levels fluctuated throughout the storage period.

**Figure 7:**
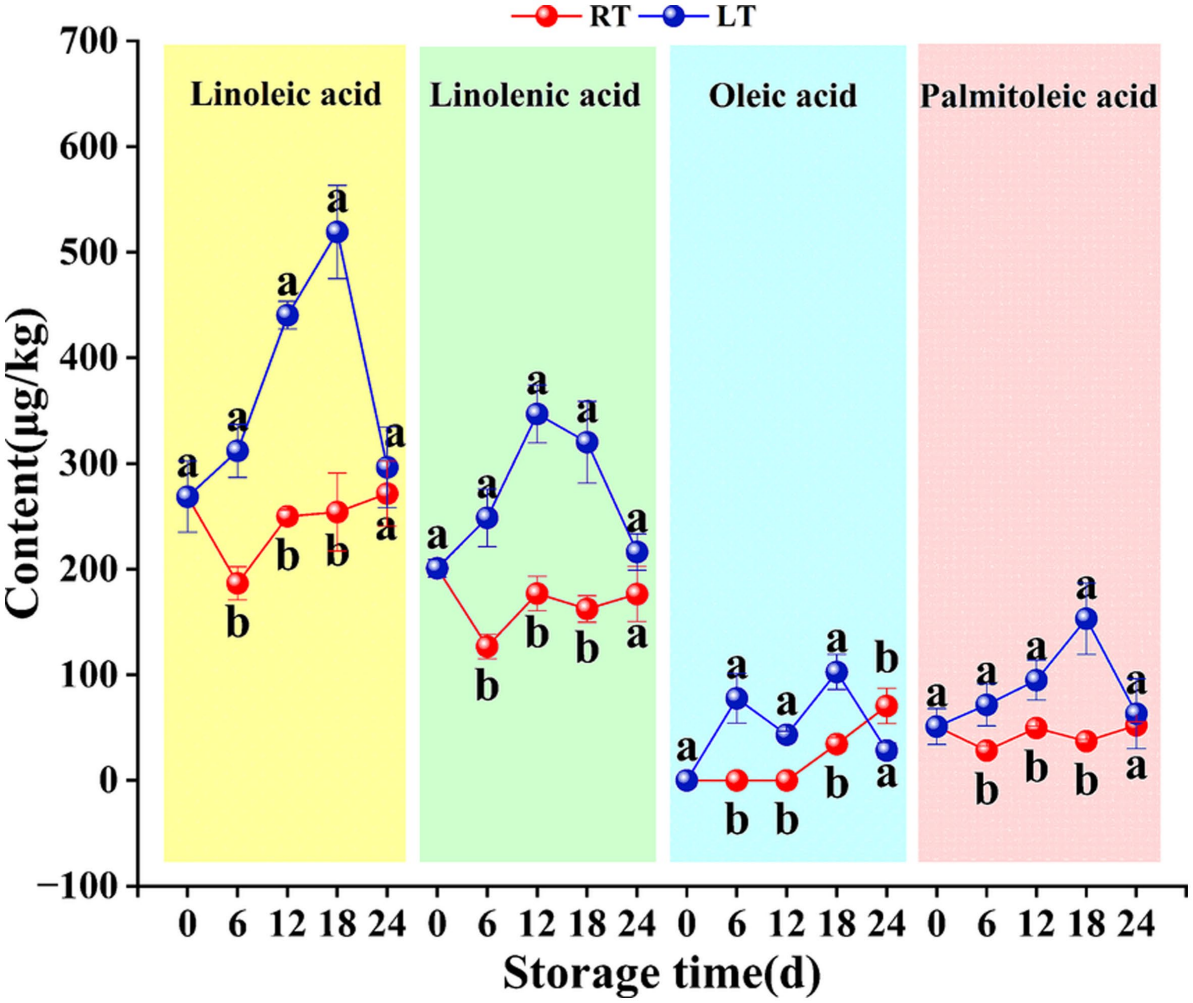
Changes in content of linoleic acid, linolenic acid, oleic acid, and palmitic acid during storage. Vertical bars represent the standard deviation (*n* = 3). Different letters on the same-day data point represent the significant differences between data (*p* < 0.05).

Notably, the unsaturated fatty acid content in Hami melon fruit under low-temperature conditions was higher than that under ambient-temperature conditions at all time points. One notable exception was oleic acid on day 24. Figure 7 clearly illustrates that the variation in unsaturated fatty acid levels was more pronounced under low-temperature conditions but limited at ambient temperature. Notably, oleic acid was not detected at ambient temperature during days 0–12 but exhibited an upward trend during days 12–24 of storage.

#### 3.4.2 Amino acid content

During the storage of Hami melons, two aromatic amino acids—phenylalanine and tyrosine—were detected, along with three branched-chain amino acids, i.e., leucine, isoleucine, and valine.

**Figure 8:**
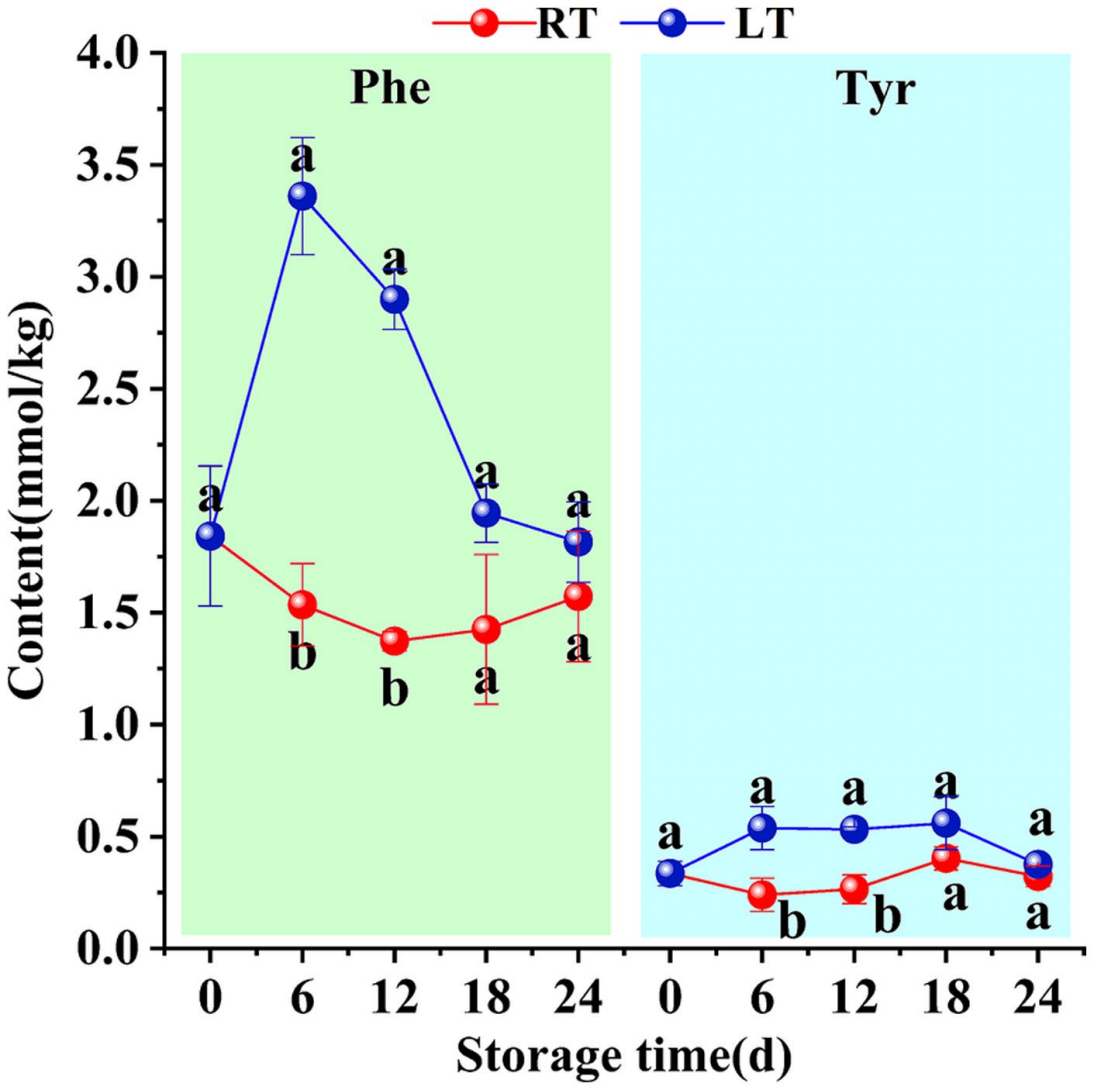
Changes in content of phenylalanine, tyrosine during storage. Vertical bars represent the standard deviation (*n* = 3). Different letters on the same-day data point represent the significant differences between data (*p* < 0.05).

Under ambient storage conditions, the phenylalanine content in Hami melon fruit initially declined before increasing thereafter. By the 12th day of storage, the concentration of this amino acid dropped to its lowest levels of 1.54 mmol/kg. Interestingly, phenylalanine levels exhibited an opposite overall trend under low-temperature conditions, peaking at 3.36 mmol/kg on the 6th day of storage. In contrast, the tyrosine content fluctuated at ambient temperature, peaking at 0.56 mmol/kg on day 18. Meanwhile, tyrosine levels remained stable between days 6 and 18 of low-temperature storage. Compared to ambient storage, low-temperature storage yielded higher phenylalanine and tyrosine levels in Hami melon fruit (Figure 8).

**Figure 9:**
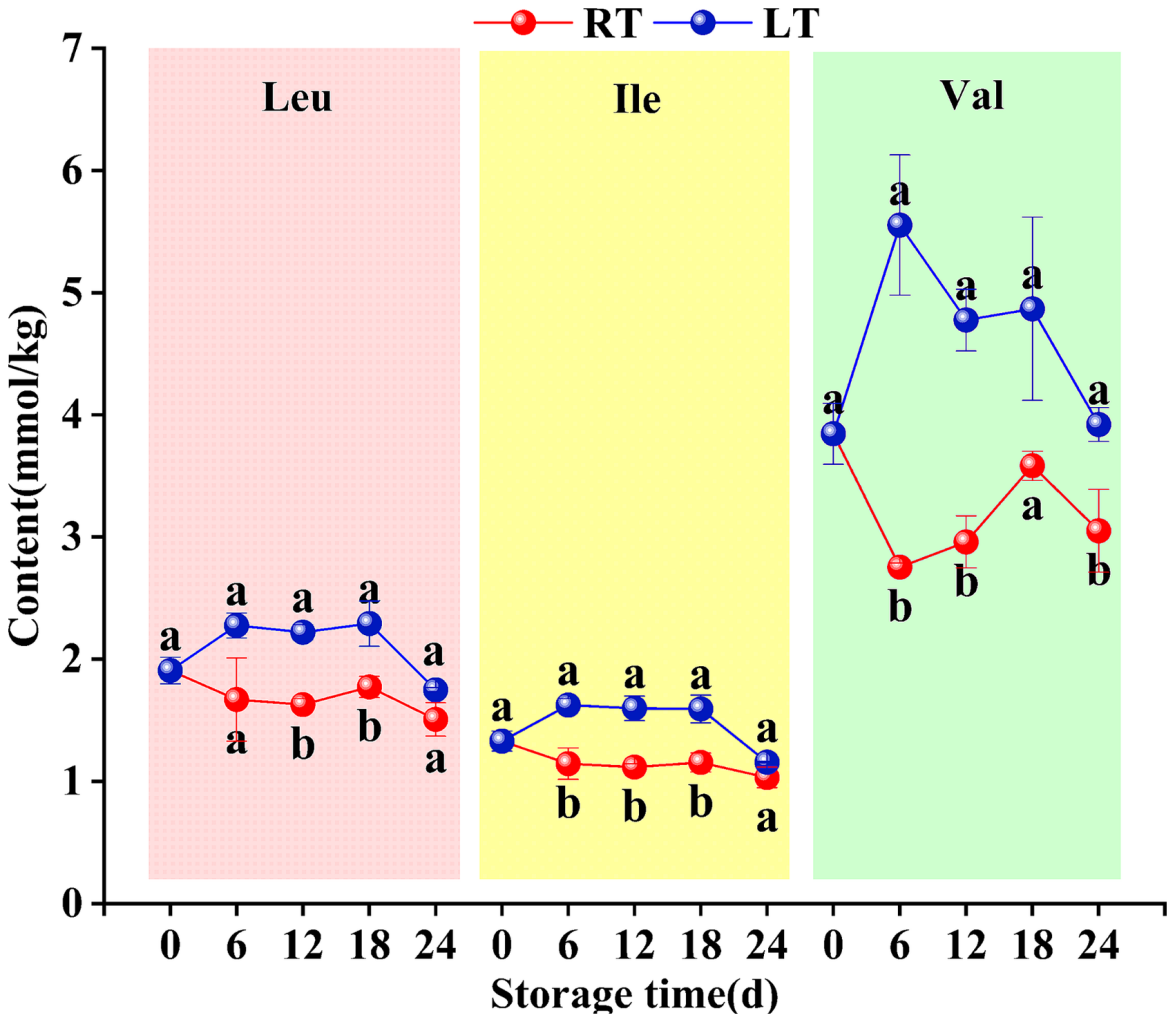
Changes in content of leucine, isoleucine, valine during storage. Vertical bars represent the standard deviation (*n* = 3). Different letters on the same-day data point represent the significant differences between data (*p* < 0.05).

During ambient storage, leucine, isoleucine, and valine levels exhibited identical trends, fluctuating in a “decline–rise–decline” pattern. All three branched-chain amino acids showed peak levels on day 18, reaching concentrations of 1.77 mmol/kg, 1.16 mmol/kg, and 3.58 mmol/kg, respectively. Under low-temperature storage conditions, the overall pattern exhibited a fluctuating trend of “rise–decline–rise–decline.” However, throughout the storage period, the levels of branched-chain amino acids in Hami melons stored at low temperature consistently exceeded those in fruit stored at ambient temperature. Furthermore, compared to leucine and isoleucine, valine showed more pronounced variation between 6 and 18 d of storage (see Figure 9).

#### 3.4.3 β-carotene content

**Figure 10:**
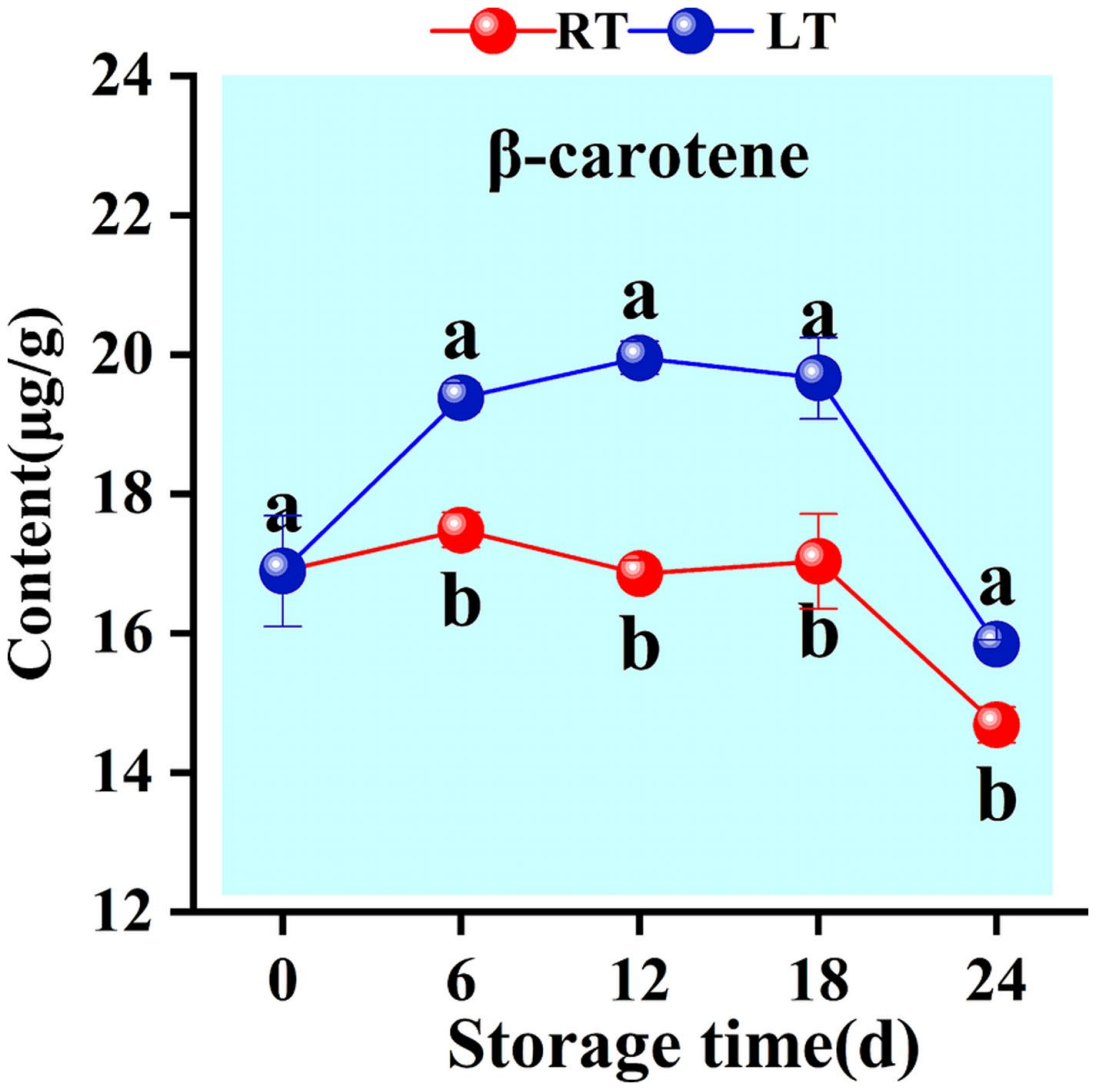
Changes in content of *β*-carotenoid content during storage. Vertical bars represent the standard deviation (*n* = 3). Different letters on the same-day data point represent the significant differences between data (*p* < 0.05).

One type of carotenoid—β-carotene—was detected during the storage of Hami melons. At ambient temperature, the β-carotene content in Hami melons exhibited a fluctuating “rise–decline–rise–decline” pattern. During the initial phase of low-temperature storage, *β*-carotene levels increased rapidly, peaking at 19.95 μg/g on day 12 before steadily declining to 15.84 μg/g, demonstrating an initial rise followed by a subsequent decrease. Throughout the storage period, the β-carotene levels under low-temperature conditions consistently exceeded those under ambient-storage conditions (Figure 10).

### 3.5 Correlation analysis of volatile odor-active compounds, key enzymes, and precursor substances

**Figure 11:**
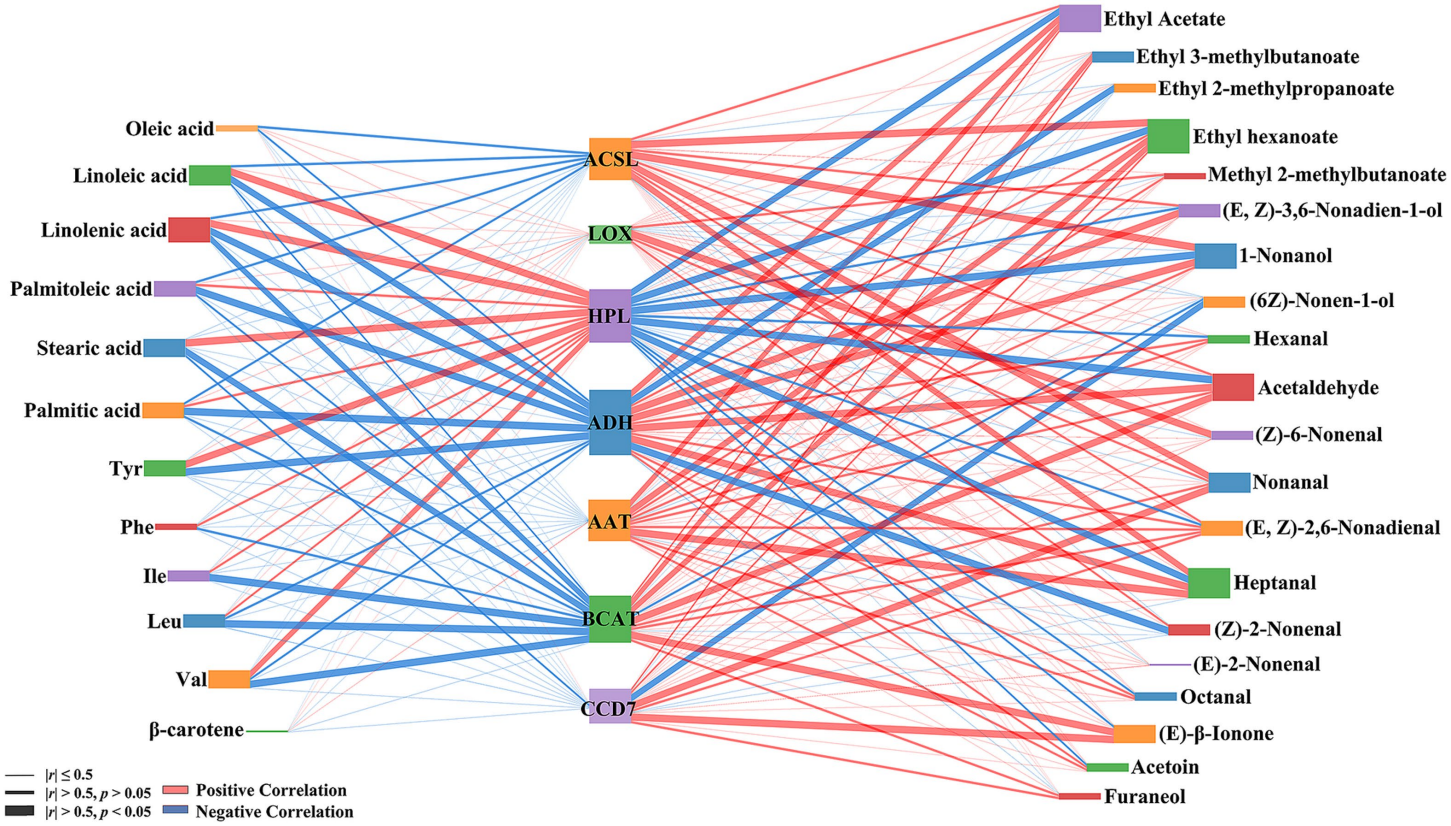
Correlation analysis of volatile odor-active compounds contents, key enzymes activities and precursors contents in cantaloupe melon during storage.

Overall, the levels of precursor substances exhibited a negative correlation with the activities of key enzymes, except LOX and HPL, while the activities of key enzymes (except HPL) showed a general positive correlation with the levels of odor-active compounds (Figure 11). ACSL activities exhibited strong negative correlations with the levels of all fatty acid precursors as well as significant positive correlations with the contents of ethyl hexanoate, 1-nonanol, nonanal, and heptanal (*p* < 0.05). LOX activities demonstrated a significant positive correlation with the levels of (Z)-6-nonenal (*p* < 0.05), while HPL activities showed significant positive correlations with the contents of linoleic acid, linolenic acid, palmitic acid, stearic acid, tyrosine, and *β*-carotene (*p* < 0.05) and significant negative correlations with the levels of ethyl acetate, ethyl hexanoate, 1-nonanol, acetaldehyde, and heptanal (*p* < 0.05). ADH activities exhibited significant negative correlations (*p* < 0.05) with the contents of linoleic acid, linolenic acid, palmitoleic acid, palmitic acid, and tyrosine. It showed significant positive correlations (*p* < 0.05) with the levels of ethyl acetate, (E,Z)-3,6-nonadien-1-ol, 1-nonanol, acetaldehyde, heptanal showed significant positive correlations (*p* < 0.05), and significant negative correlations with the contents of ethyl 2-methylpropanoate and (Z)-2-nonenal (*p* < 0.05). Meanwhile, AAT activities showed significant positive correlations with the levels of ethyl acetate, ethyl hexanoate, and heptanal (*p* < 0.05). In contrast, BCAT activities exhibited significant negative correlations with the contents of linolenic acid, stearic acid, and branched-chain amino acids (*p* < 0.05) as well as significant positive correlations with the levels of ethyl 3-methylbutyrate, ethyl hexanoate, acetaldehyde, and *β*-ionone (*p* < 0.05). Finally, CCD7 activities showed a significant negative correlation with the contents of (6Z)-non-1-enol (*p* < 0.05) and a significant positive correlation with the levels of nonanal and β-ionone (*p* < 0.05).

## 4 Discussion

### 4.1 Effects of low-temperature storage on aroma-related fatty acid pathways

Fatty acids serve as the primary precursors for most volatile fruit aromatics (25). The straight-chain alcohols, aldehydes, esters, and lactones produced from fatty acids act as the key characteristic odor-active compounds of fresh fruit, collectively shaping their flavour profile (26, 27). The key biosynthetic enzymes involved in the fatty acid LOX pathway include LOX, HPL, ADH, and AAT (28–31). Meanwhile, ACSL is responsible for activating free fatty acids, thereby supplying substrates for the LOX pathway (32).

In this study, the saturated fatty acids palmitic acid and stearic acid exhibited higher concentrations at low storage temperatures than at ambient temperature (Figure 6), consistent with findings in southern pears (33). In fruit, saturated fatty acids are broken down into shorter-chain acyl-CoA molecules via *β*-oxidation, which are then reduced by acyl-CoA reductase into aldehyde compounds. Subsequently, through the action of ADH and AAT, these aldehydes are converted into alcohols and ultimately into esters (28, 30, 34). Given the continuous rise in linoleic and linolenic acid levels detected during the early storage period under low-temperature conditions (Figure 7), it appears that during this phase, low temperatures inhibit the β-oxidation of saturated fatty acids in Hami melons, leading to a certain degree of saturated fatty acid accumulation. However, stearic acid may partially be converted into unsaturated fatty acids via the LOX pathway through the fatty acid desaturase FAD or contribute to aroma metabolism through other pathways, thereby generating volatile compounds.

Unsaturated fatty acids are converted to C6 aldehydes by LOX, and these compounds are then converted into alcohols via ADH. The alcohols undergo acetylation to produce esters in reactions catalysed by AAT (29, 31). In this study, at ambient temperature, LOX enzyme activity reached its peak between 0 and 6 d (Figure 2). This led to the oxidative degradation of precursor substances such as linoleic acid and linolenic acid. Consequently, the content of linoleic acid, linolenic acid, and palmitic acid exhibited a decreasing trend (Figure 7). Meanwhile, the content of characteristic C6 and C9 odor-active compounds, including (E,Z)-3,6-nonadien-1-ol, hexanal, (Z)-6-nonenal, and (E,Z)-2,6-nonadienal, showed a marked increase (Supplementary Table S2). Tang et al. (35) demonstrated that unsaturated fatty acids such as linoleic acid and linolenic acid, acting as LOX substrates, enhance LOX activity in melon fruit and promote the accumulation of compounds such as hexanal, (E)-2-nonenal, and ethyl acetate. Meanwhile, the present study also showed that low-temperature storage inhibits the oxidation and cleavage of unsaturated fatty acids in melons by suppressing the activity of key enzymes such as ACSL and LOX. This causes the significant accumulation of unsaturated fatty acids when compared to ambient-temperature storage, consistent with previous findings in Nanguo pears (H. (9, 11)).

### 4.2 Effects of low-temperature storage on aroma-related amino acid pathways

Amino acids are not only important nutrients but also act as precursors for the synthesis of odor-active compounds in fruit (36–40). The amino acids involved in the synthesis of volatile compounds can primarily be categorised into two groups: aromatic amino acids (phenylalanine and tyrosine) and branched-chain amino acids (leucine, isoleucine, and valine) (41, 42).

Phenylalanine serves as a precursor for the synthesis of various odor-active compounds, influencing the overall aroma characteristics of fruit (43). In this study, under low-temperature storage conditions, both phenylalanine and tyrosine levels in Golden Queen Hami melons were found to be higher than those at ambient temperature (Figure 8), indicating that low temperatures promote phenylalanine accumulation (44). Furthermore, phenylalanine exhibited a marked decrease between 6 and 18 d, whilst tyrosine showed a slight increase. This could be attributed to the direct conversion of phenylalanine into tyrosine via hydroxylation.

In plants, branched-chain amino acids serve as the primary source of volatile compounds such as esters, aldehydes, and alcohols (41). BCAT is one key enzyme involved in the generation of odor-active compounds through amino acid metabolism (45). In this study, during storage at ambient temperature, BCAT enzyme activity in melon fruit was found to exhibit an overall upward trend (Figure 4), consistent with previous findings (46). Correlation analysis revealed the significant negative correlations between BCAT activity and leucine, valine, and isoleucine levels (Figure 11), in line with its ability to catalyse the transamination of these amino acids into their respective *α*-keto acids (47). Ethyl 3-methylbutyrate, derived from leucine, exhibited peak concentrations after 18 d of ambient-temperature storage, coinciding with peak BCAT activity levels. Methyl 2-methylbutyrate, derived from isoleucine, showed its highest concentration after 12 d at ambient temperature, and this was accompanied by the depletion of its precursor and the increase in BCAT activity. In contrast, ethyl 2-methylpropanoate, derived from valine, was only detected after 18 d at low temperature. Gonda et al. (45) found that exogenous L-leucine, exogenous L-isoleucine, and L-valine increased the levels of 3-methylbutanol derivatives, 2-methylbutanol derivatives, and 2-methylpropanol derivatives in melon fruit, respectively. These findings further corroborate the conversion of branched-chain amino acids into downstream aroma derivatives under the action of BCAT.

### 4.3 Effects of low-temperature storage on aroma-related Terpenoid synthesis pathways

Carotenoids, produced via terpenoid synthesis pathways, are considered key components of fruit flavour profile (48). Carotenoids are the fundamental compounds responsible for the vibrant yellow colouration of Golden Queen Hami melons (49, 50). As one of the primary carotenoids in fruit pulp, maintaining *β*-carotene levels may help delay the deterioration of pulp colour. β-carotene accumulates slowly in this fruit during the early storage period, with its levels increasing rapidly between 6 d and 18 d (Figure 10). This trend is consistent with previous findings from peach fruit (51). Notably, the present study indicated that the downregulation of CCD7-related genes during low-temperature storage (Figure 5) leads to reduced CCD7 activity, resulting in β-carotene accumulation, in line with previous research on nectarines (52). Further evidence also confirmed that low-temperature conditions have a positive influence on β-carotene accumulation. β-carotene can undergo oxidative cleavage in a reaction catalysed by CCD7 to yield β-cryptoxanthin (53, 54). In this study, β-ionone persisted throughout storage and exhibited a significant positive correlation with CCD7 activity (Figure 11), contributing substantially to the aroma profile of Hami melons (Table 1). Evidence from previous studies (55, 56) also corroborates these results.

Overall, the present study demonstrated that the post-harvest aroma quality of Hami melons is regulated by multiple pathways, including fatty acid degradation and oxidation, amino acid degradation and conversion, and terpenoid biosynthesis. These pathways comprise a complex network through which various precursor substances, such as fatty acids, amino acids, and carotenoids, are enzymatically converted into odor-active compounds. Notably, the evidence shows that low-temperature storage may be more effective at preserving melon flavour quality by controlling overall metabolic activity in the fruit and reducing precursor consumption.

## 5 Conclusion

This study demonstrates that low-temperature storage inhibits the activity of enzymes such as ACSL, BCAT, ADH, AAT, and CCD7 in Golden Queen Hami melons by regulating the expression of genes encoding key enzymes involved in odor-active compounds metabolism. This significantly reduces the consumption of fatty acids (linoleic acid and palmitic acid), amino acids (phenylalanine and valine), and β-carotene, leading to their accumulation under low-temperature conditions. Concurrently, the content of primary odor-active compounds such as esters, alcohols, and aldehydes also decreases. Through correlation analysis, this study reveals that key enzymes, acting as pivotal nodes, predominantly exhibit negative correlations with the levels of precursor substances while generally showing positive correlations with the content of odor-active compounds. These findings not only provide theoretical support for optimising low-temperature storage techniques for post-harvest melon fruit but also offer novel insights into the aroma metabolic pathways that can be targeted to enhance fruit flavour.

## Data Availability

The original contributions presented in the study are included in the article/Supplementary material, further inquiries can be directed to the corresponding authors.
