# Supplementary material for: Aroma metabolism during the low-temperature storage of Hami melons: volatile odor-active compounds, precursors, and enzyme activity
Source: Front Nutr. 2026 Mar 30;13:1775610. doi: 10.3389/fnut.2026.1775610 (PMC13070788; doi:10.3389/fnut.2026.1775610)
Supplement: Supplementary file 1 [file Table_1.docx]

| **Gene ID** | **Forward primer (5′to 3′)** | **Reverse primer (5′to 3′)** | **Product (bp)** |
| --- | --- | --- | --- |
| *ACSL*(LOC103482821) | TGGTTCAGGTGGAGGAGGCTAAG | CTACTTTCCTACGCCCAAGCATCC | 186 |
| *LOX*(LOC10348906) | TTACAGCAACACTTCGAGTCATCCG | GCAGCAGTCTCCACAGTCTTCTTC | 170 |
| *HPL*(LOC103495615) | CATCCACATCGGCGTTCTCCAG | TCTTTGGTAGCGGGAAGCAATGG | 91 |
| *ADH*(LOC103482770) | AGGGATGTGATTGTCCTGTGATTGG | CTCAACAACACCTGATGCTTCATGG | 195 |
| *BCAT*(LOC103484883) | ACCGTCAGTCTATCGTAGGCATAGG | ATCTCTCGGGACTGGCAGAAAGG | 73 |
| *CCD7*(LOC103498824) | GCTGAAGGGAGGGAAGAAATTAGGG | ACCGAAGCACACTCGTATTGGC | 71 |

**Supplementary Table 1 The sequences of speciﬁc primers used for qRT-PCR analysis**

**Supplementary Table 2 Tentative identification of odor-active compounds during storage**

| **Number** | **Compounds** | **RT** | | | | | **LT** | | | | | **Fragrance Type** |
| --- | --- | --- | --- | --- | --- | --- | --- | --- | --- | --- | --- | --- |
|  |  | **0d** | **6d** | **12d** | **18d** | **24d** | **0d** | **6d** | **12d** | **18d** | **24d** |  |
| A1 | (E)-3-Hexenyl Acetate | ND | ND | 5.54±0.8 | ND | ND | ND | ND | 0.29±0.02 | 0.11±0.04 | 0.11±0.04 | fruity |
| A2 | Dimethyl phthalate | 3.62±0.66 | 1.33±0.58 | ND | 1.98±0.04 | 5.08±0.88 | 3.62±0.66 | ND | ND | ND | ND | - |
| A3 | Isobutyl acetate | ND | 4.77±0.4 | ND | ND | ND | ND | ND | ND | ND | ND | fruity |
| A4 | Methyl Benzoate | 2.89±0.19 | 4.25±0.67 | ND | 0±0 | ND | 2.89±0.19 | ND | 0.95±0.08 | ND | 1.06±0.91 | phenolic |
| A5 | Ethyl Acetate | 116.18±1.91 | 258.25±25.62 | 197.88±18.81 | 111.15±11.78 | 110.3±11.55 | 116.18±1.91 | ND | ND | 0±0 | ND | ethereal |
| A6 | Methyl Butyrate | ND | 1.3±0.61 | ND | ND | ND | ND | ND | ND | ND | ND | fruity |
| A7 | Ethyl 3-methylbutanoate | ND | 0.21±0.01 | ND | 1.85±0.26 | ND | ND | ND | ND | ND | ND | fruity |
| A8 | Ethyl Isovalerate | ND | ND | ND | ND | 1.92±0.14 | ND | ND | ND | ND | ND | waxy |
| A9 | Hexyl Acetate | ND | 2.44±0.51 | ND | ND | ND | ND | ND | ND | ND | ND | fruity |
| A10 | Methyl Hexanoate | ND | 0.26±0.06 | ND | ND | ND | ND | ND | ND | ND | ND | fruity |
| A11 | Heptyl Formate | ND | ND | 0.75±0.43 | ND | ND | ND | ND | ND | ND | ND | green |
| A12 | 2-Methylbutyl Acetate | 1.4±1.05 | 4.3±0.61 | 2.75±0.42 | ND | ND | 1.4±1.05 | ND | ND | 0.46±0.15 | ND | fruity |
| A13 | (E)-2-Hexenyl Acetate | ND | 3.07±0.89 | 0.73±0.47 | ND | ND | ND | ND | ND | ND | ND | green |
| A14 | Ethyl Propionate | ND | ND | ND | ND | ND | ND | ND | ND | 5.52±0.83 | ND | fruity |
| A15 | Methyl Phenylacetate | 60.06±0.91 | 25.23±2.66 | 12.14±1.8 | ND | ND | 60.06±0.91 | ND | ND | 0.54±0.28 | 1.31±0.6 | floral |
| A16 | Ethyl 2-methylpropanoate | ND | ND | ND | ND | ND | ND | ND | ND | 2.2±0.72 | ND | fruity |
| A17 | 2-Phenethyl Hexanoate | ND | 0.05±0.03 | ND | ND | 1.53±0.81 | ND | 3.26±0.65 | 0.1±0.05 | 0.24±0.07 | ND | floral |
| A18 | 2-Ethylbutyl Acetate | ND | 1.31±0.6 | ND | ND | ND | ND | ND | ND | ND | ND | fruity |
| A19 | Ethyl hexanoate | 2.26±1.28 | 9.62±0.65 | 7.04±0.94 | 4.97±0.06 | 3.75±0.44 | 2.26±1.28 | 1.24±0.67 | 2.35±0.56 | 0.94±0.11 | 0.05±0.03 | fruity |
| A20 | Methyl Acetate | ND | 6.65±0.6 | 0±0 | ND | ND | ND | ND | 0±0 | ND | ND | ethereal |
| A21 | 2-Butoxyethyl acetate | 24.84±0.27 | ND | ND | ND | ND | 24.84±0.27 | ND | ND | ND | ND | - |
| A22 | α-Angelica Lactone | ND | 41.9±3.86 | ND | ND | ND | ND | ND | ND | ND | ND | coconut |
| A23 | Methyl Palmitate | ND | ND | ND | ND | 1.79±0.37 | ND | ND | ND | ND | ND | waxy |
| A24 | Methyl 2-methylbutanoate | ND | 2.18±0.75 | ND | ND | ND | ND | ND | 0.72±0.48 | ND | 0.22±0.07 | fruity |
| A | Esters | 211.25±3.93 | 367.12±36.82 | 226.83±21.74 | 119.94±10.91 | 124.37±12.45 | 211.25±3.93 | 4.5±0.5 | 4.42±0.52 | 10.01±1.99 | 2.74±0.45 |  |
| B1 | 2-methyl-5-(1-methylethenyl)-Cyclohexanol | ND | ND | ND | ND | ND | ND | ND | ND | ND | 0.05±0.93 | minty |
| B2 | (E)-2-Hepten-1-ol | 0.49±0.18 | ND | ND | ND | ND | 0.49±0.18 | ND | 1.60±0.68 | 0.54±0.79 | 2.57±0.74 | fatty |
| B3 | (E)-2-Octen-1-ol | 0.49±0.18 | 2.23±0.68 | 1.58±0.73 | 0.91±0.15 | 0.29±0.02 | 0.49±0.18 | 0±0 | 0±0 | ND | ND | green |
| B4 | (E,Z)-3,6-Nonadien-1-ol | 135.37±1.49 | 268.43±26.36 | 116.11±11.84 | 176.2±17.7 | 165.05±16.92 | 135.37±1.49 | 231.67±22.51 | 52.04±5.95 | 54.00±6.00 | 117.62±10.43 | green |
| B5 | 2-Nonen-1-ol | ND | 0.89±0.19 | 1.53±0.81 | 8.69±0.54 | 0.16±0.07 | ND | 0.80±0.34 | 0.21±0.11 | 2.13±0.82 | ND | fatty |
| B6 | 2-Methyl-1-Butanol | ND | ND | ND | 2.32±0.59 | ND | ND | ND | ND | ND | ND | ethereal |
| B7 | (Z)-4-Hexen-1-ol | 0.51±0.04 | ND | 0.09±0.06 | ND | ND | 0.51±0.04 | ND | ND | 0.60±0.09 | 0.49±0.15 | green |
| B8 | Phenylethyl Alcohol | ND | ND | ND | 5.92±0.14 | 6.49±0.50 | ND | 1.22±0.70 | ND | ND | 0.44±0.51 | floral |
| B9 | 1-Nonanol | 59.81±0.74 | 78.97±6.96 | 57.35±5.48 | 54.14±5.79 | 43.65±3.49 | 59.81±0.74 | 42.75±3.63 | 18.35±1.51 | 15.98±0.97 | 31.68±2.53 | floral |
| B10 | (Z)-3-Nonen-1-ol | 200.31±2.33 | 484.22±48.67 | 429.42±42.37 | 338.99±32.99 | 333.19±33.71 | 200.31±2.33 | 207.15±20.78 | 118.38±11.43 | 100.92±9.88 | 151.95±14.93 | waxy |
| B11 | 2-Furanmethanol | ND | ND | 146.47±14.3 | ND | ND | ND | ND | ND | ND | ND | bready |
| B12 | Benzyl alcohol | 24.11±0.83 | ND | ND | ND | ND | 24.11±0.83 | ND | ND | ND | ND | floral |
| B13 | 1-Hexanol | ND | 1.48±0.50 | ND | ND | 1.44±0.51 | ND | ND | 2.11±0.84 | 1.70±0.52 | 1.11±0.85 | herbal |
| B14 | 3,7-dimethyl-1,7-Octanediol | ND | 0.48±0.25 | ND | ND | ND | ND | ND | ND | ND | 0.06±0.01 | floral |
| B15 | 4-Hexen-1-ol | 0.24±0.11 | ND | ND | ND | ND | 0.24±0.11 | ND | ND | ND | ND | green |
| B16 | (6Z)-Nonen-1-ol | 21.78±0.39 | 11.43±1.41 | 12.37±1.48 | 0.70±0.52 | ND | 21.78±0.39 | ND | 12.56±0.51 | 9.64±0.63 | 20.69±1.55 | melon |
| B | Alcohols | 443.11±3.56 | 848.12±84.81 | 764.92±75.88 | 587.87±57.81 | 550.27±55.6 | 443.11±3.56 | 483.59±47.39 | 205.25±20.62 | 185.5±17.26 | 326.65±31.48 |  |
| C1 | β-Cyclocitral | ND | ND | ND | ND | ND | ND | ND | 0.21±0.71 | ND | 0.46±0.05 | tropical |
| C2 | Hexanal | 0.24±1.31 | 85.3±8.55 | 4.92±0.15 | 14.57±0.51 | 0.61±0.68 | 0.24±1.31 | 1.73±0.46 | 5.29±0.61 | 1.77±0.39 | 4.04±0.94 | green |
| C3 | Acetaldehyde | 40.34±0.57 | 111.14±11.79 | 39.42±3.39 | 70.29±7.57 | 50.70±4.55 | 40.34±0.57 | 25.27±2.60 | 9.23±0.68 | 18.56±0.51 | 26.22±2.68 | ethereal |
| C4 | Pentanal | ND | ND | 6.17±0.76 | ND | ND | ND | ND | ND | ND | 4.18±0.75 | fermented |
| C5 | 5-heptyldihydro-2(3H)-Furanone | 0.36±0.11 | ND | ND | ND | 1.75±0.43 | 0.36±0.11 | 0.42±0.22 | ND | ND | ND | fruity |
| C6 | (Z)-6-Nonenal | 3.00±0.99 | 57.09±5.87 | 31.99±2.99 | 8.36±0.56 | 21.5±2.29 | 3.00±0.99 | ND | 15.37±1.48 | 9.31±0.60 | 26.25±2.64 | melon |
| C7 | Dodecanal | ND | ND | ND | ND | ND | ND | ND | 0.03±0.95 | ND | ND | aldehydic |
| C8 | Nonanal | 0.12±1.52 | 50.42±5.38 | 82.08±8.88 | 48.88±3.82 | 20.79±1.7 | 0.12±1.52 | 6.74±0.46 | 16.63±0.55 | 6.02±0.97 | 23.98±1.97 | aldehydic |
| C9 | (E)-2-Decenal | ND | ND | ND | 2.13±0.81 | ND | ND | ND | 0.73±0.48 | 0.51±0.84 | ND | fatty |
| C10 | (E)-2-Hexenal | ND | 1.39±0.54 | ND | ND | ND | ND | ND | 0.10±0.85 | 0.13±0.81 | 0.11±0.85 | green |
| C11 | (E,E)-2,4-Nonadienal | ND | ND | ND | ND | ND | ND | ND | 0.04±0.01 | ND | ND | fatty |
| C12 | (E,Z)-2,6-Nonadienal | 5.47±0.92 | 141.58±13.37 | 127.75±11.63 | 17.6±0.53 | 114.9±10.85 | 5.47±0.92 | 12.05±1.93 | 20.04±2.95 | 6.13±0.81 | 22.94±1.92 | green |
| C13 | Heptanal | 3.58±0.73 | 6.33±0.58 | 5.32±0.59 | 2.5±0.86 | 0.63±0.44 | 3.58±0.73 | 0.75±0.43 | ND | 0.12±0.03 | 0.30±0.01 | green |
| C14 | (E)-2-Octenal | ND | ND | ND | ND | ND | ND | ND | 1.17±0.76 | ND | 0.24±0.07 | fatty |
| C15 | 5-methyl-2-Furancarboxaldehyde | ND | ND | 30.3±3.56 | ND | ND | ND | ND | ND | ND | ND | caramellic |
| C16 | Benzaldehyde | 91.57±0.52 | 59.97±4.96 | 16.73±0.64 | 9.44±0.51 | 22.86±1.80 | 91.57±0.52 | 16.66±0.57 | 10.87±0.81 | 13.11±1.84 | 13.17±1.74 | fruity |
| C17 | Decanal | 1.00±0.75 | ND | 0.16±0.07 | ND | 0.72±0.48 | 1.00±0.75 | 1.40±0.53 | 1.27±0.64 | 0.7±0.52 | 1.36±0.56 | aldehydic |
| C18 | (Z)-2-Nonenal | ND | ND | 0.86±0.24 | ND | ND | ND | ND | 1.30±0.61 | 2.40±0.53 | ND | fatty |
| C19 | (E)-2-Nonenal | ND | ND | 115.8±10.71 | ND | 95.95±8.93 | ND | 6.69±0.53 | 25.24±2.65 | 4.91±0.16 | 22.22±2.67 | fatty |
| C20 | Octanal | ND | 4.64±0.63 | 0.32±0.18 | ND | 1.3±0.61 | ND | ND | ND | ND | ND | aldehydic |
| C | Aldehydes | 145.69±1.14 | 517.86±50.79 | 461.82±45.73 | 173.77±16.66 | 331.72±32.58 | 145.69±1.14 | 71.71±6.57 | 107.53±9.30 | 63.68±5.53 | 145.46±14.31 |  |
| D1 | 6-Methyl-5-Hepten-2-one | ND | 3.36±0.56 | 2.38±0.54 | 4.02±0.97 | 2.45±0.51 | ND | 0.59±0.32 | 0.66±0.58 | 1.09±0.87 | 2.56±0.76 | citrus |
| D2 | (E)-β-Ionone | 0.15±1.47 | 1.40±0.53 | 0.99±0.02 | 3.04±0.94 | 1.46±0.50 | 0.15±1.47 | 0.32±0.19 | 0.52±0.13 | 0.40±0.23 | 0.54±0.09 | floral |
| D3 | Acetoin | ND | 19.47±1.36 | ND | ND | ND | ND | ND | ND | ND | ND | buttery |
| D4 | 2-Octanone | 3.01±0.98 | 5.1±0.86 | 0.24±0.08 | ND | 2.04±0.95 | 3.01±0.98 | 3.52±0.83 | 4.27±0.64 | 2.66±0.59 | 2.97±0.05 | earthy |
| D5 | (E)- 6,10-dimethyl-5,9-Undecadien-2-one | 2.67±0.57 | 13.13±1.81 | 1.72±0.48 | 5.61±0.68 | 11.1±1.85 | 2.67±0.57 | ND | 4.12±0.82 | 4.83±0.30 | 6.89±0.19 | floral |
| D6 | 2-Pentanone | ND | ND | 8.57±0.75 | ND | ND | ND | ND | ND | ND | ND | fruity |
| D7 | Furaneol | ND | ND | ND | 36.74±2.61 | ND | ND | ND | ND | ND | ND | caramellic |
| D | Ketones | 5.84±0.28 | 42.46±4.33 | 13.89±0.85 | 49.4±4.42 | 17.05±1.92 | 5.84±0.28 | 4.43±0.51 | 9.58±0.74 | 8.97±0.05 | 12.96±0.94 |  |

The values in the table represent the compound content.
